# 69. Incidence of metabolic complications among treatment-naïve adults living with HIV-1 randomized to B/F/TAF, DTG/ABC/3TC or DTG+F/TAF after 144 Weeks

**DOI:** 10.1093/ofid/ofab466.069

**Published:** 2021-12-04

**Authors:** Eric Daar, Chloe Orkin, Paul Sax, Jeffrey L Stephens, Ellen Koenig, Amanda Clarke, Axel Baumgarten, Cynthia Brinson, Moti Ramgopal, Hailin Huang, Terry Farrow, Jared Baeten, Jason Hindman, Hal Martin, Kimberly Workowski

**Affiliations:** 1 The Lundquist Institute, Torrance, California; 2 Barts Health NHS Trust, Royal London Hospital, Ambrose King Centre, London, England, United Kingdom; 3 Brigham and Women's Hospital, Boston, MA; 4 Mercer University School of Medicine, Macon, GA; 5 Instituto Dominicano de Estudio Virologicos – IDEV, Santo Domingo, Distrito Nacional, Dominican Republic; 6 University Hospitals Sussex NHS Foundation, London, England, United Kingdom; 7 Zentrum für Infektiologie Berlin Prenzlauer Berg, Berlin, Brandenburg, Germany; 8 Central Texas Clinical Research, Austin, Texas; 9 Midway Specialty Care Centers, Fort Pierce, Florida; 10 Gilead Sciences Inc., Foster City, California; 11 Emory University, Atlanta, GA

## Abstract

**Background:**

Metabolic comorbidities including diabetes (DM) and dyslipidemia pose challenges to the long-term care of people with HIV (PWH). Incidence of cardiovascular disease and DM are reported at higher rates in PWH than the general population. Obesity is broadly prevalent in both the general population and PWH, and higher body mass index (BMI) can contribute to metabolic complications. Here we present longer-term follow up on incidence of DM, hypertension (HTN), BMI categorical shifts, and lipid changes over 144 weeks of blinded treatment from two trials of PWH initiating antiretroviral therapy.

**Methods:**

We assessed incidence of metabolic complications in adult PWH in Study 1489: bictegravir/emtricitabine/tenofovir alafenamide (B/F/TAF) vs dolutegravir/abacavir/ lamivudine (DTG/ABC/3TC) and Study 1490: B/F/TAF vs DTG+F/TAF. Treatment-emergent (TE) metabolic comorbidities were defined by standard MedDRA search lists. CDC-defined BMI categories were compared from baseline (BL) to Week 144. Analyses by sex at birth and race were performed, as well as for lipid changes.

**Results:**

Among 1,274 total participants, median (range) age was 33 years (18-77), 90% men, 33% black. In study 1489, BL prevalence of DM and HTN was 4.5 and 12.1% with TE DM and HTN in B/F/TAF being 0.7% and 10%, and for DTG/ABC/3TC 1.3% and 6.9%, respectively. In study 1490, BL prevalence of DM and HTN was 6.8 and 18.8% with TE DM and HTN in B/F/TAF being 2.1 and 5.8%, and for DTG+F/TAF 2.3 and 6.5%, respectively. BMI shift from Normal to Obese: B/F/TAF 0%, DTG/ABC/3TC 3.2%, p=0.12 (1489) (Table 1); B/F/TAF 2.5%, DTG+F/TAF 2.9% p=1.00 (1490) (Table 2). Subgroup analyses by gender/race showed similar findings for TE DM, HTN, and BMI changes. Median changes from BL fasted lipids were small (Table 1).

Table 1§. Studies 1489 and 1490: Metabolic Outcomes from Baseline to Week 144

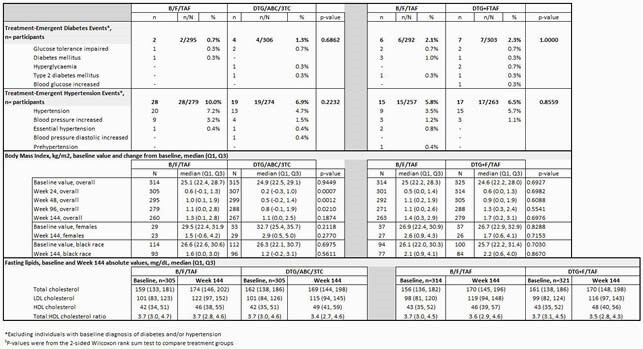

Table 2±. Shift Table of BMI Category at Week 144 by Baseline BMI Category – Overall

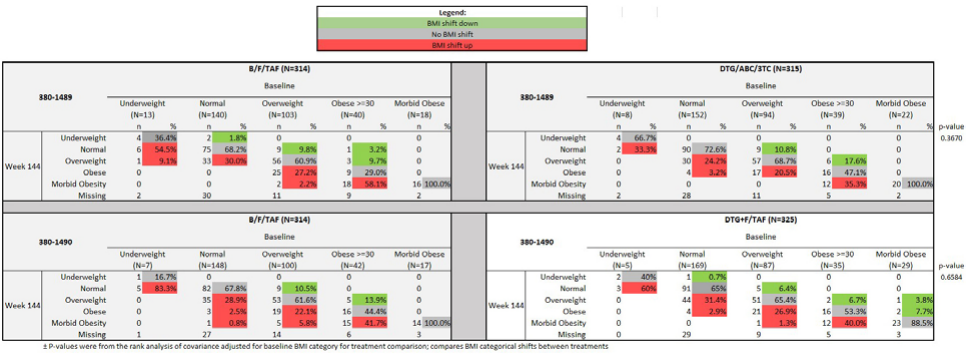

**Conclusion:**

Through over 144 weeks of follow up, PWH randomized to initiate B/F/TAF, DTG/ABC/3TC or DTG+F/TAF had low rates of incident DM or HTN-related AEs, with no statistically significant differences by treatment group. BMI changes/categorical shifts from BL did not significantly differ by regimen, and no clinically significant change or difference by regimen in lipids were observed. While data are limited by three years of follow up, they are strengthened by randomized study design of three widely used initial ART regimens.

**Disclosures:**

**Eric Daar, MD**, **Bristol-Myers Squibb** (Consultant)**Gilead Sciences Inc.** (Consultant, Scientific Research Study Investigator, Advisor or Review Panel member, Research Grant or Support)**Janssen** (Consultant, Advisor or Review Panel member, Research Grant or Support)**Merck** (Consultant, Advisor or Review Panel member, Research Grant or Support)**Teva** (Consultant, Advisor or Review Panel member)**ViiV Healthcare** (Consultant, Advisor or Review Panel member, Research Grant or Support) **Chloe Orkin, MD**, **Gilead Sciences Inc.** (Grant/Research Support, Scientific Research Study Investigator, Other Financial or Material Support)**Janssen** (Research Grant or Support, Other Financial or Material Support)**Merck** (Research Grant or Support, Other Financial or Material Support)**ViiV Healthcare** (Research Grant or Support, Other Financial or Material Support) **Paul Sax, MD**, **Gilead Sciences** (Consultant, Grant/Research Support)**Janssen** (Consultant)**Merck** (Consultant, Research Grant or Support)**ViiV** (Consultant, Research Grant or Support) **Jeffrey L. Stephens, MD**, **Gilead Sciences Inc.** (Scientific Research Study Investigator, Research Grant or Support) **Ellen Koenig, MD**, **Gilead Sciences Inc.** (Scientific Research Study Investigator) **Amanda Clarke, MD**, **Gilead Sciences Inc.** (Consultant, Scientific Research Study Investigator, Other Financial or Material Support, Conference attendance sponsorship)**ViiV Healthcare** (Consultant, Other Financial or Material Support, Conference travel sponsorship) **Axel Baumgarten, MD**, **AbbVie** (Advisor or Review Panel member, Speaker’s Bureau)**Bristol-Myers Squibb** (Advisor or Review Panel member, Speaker's Bureau)**Gilead Sciences Inc.** (Scientific Research Study Investigator, Advisor or Review Panel member, Speaker's Bureau)**Janssen** (Speaker’s Bureau)**Merck** (Advisor or Review Panel member) **Cynthia Brinson, MD**, **Abbvie** (Scientific Research Study Investigator)**BI** (Scientific Research Study Investigator)**Gilead Sciences Inc.** (Scientific Research Study Investigator, Advisor or Review Panel member, Speaker's Bureau, Personal fees)**GSK** (Scientific Research Study Investigator)**Novo Nordisk** (Scientific Research Study Investigator)**ViiV Healthcare** (Scientific Research Study Investigator, Advisor or Review Panel member, Speaker's Bureau) **Moti Ramgopal, MD FIDSA**, **Abbvie** (Scientific Research Study Investigator, Speaker's Bureau)**Gilead** (Consultant, Scientific Research Study Investigator, Speaker's Bureau)**Janssen** (Consultant, Scientific Research Study Investigator, Research Grant or Support, Speaker's Bureau)**Merck** (Consultant, Scientific Research Study Investigator)**ViiV** (Consultant, Scientific Research Study Investigator, Speaker's Bureau) **Hailin Huang, PhD**, **Gilead Sciences Inc.** (Employee, Shareholder) **Terry Farrow, MD**, **Gilead Sciences Inc.** (Employee, Shareholder) **Jared Baeten, MD, PHD**, **Gilead Sciences Inc.** (Employee, Shareholder) **Jason Hindman, PharmD**, **Gilead Sciences Inc.** (Employee, Shareholder) **Hal Martin, MD, MPH**, **Gilead Sciences Inc.** (Employee, Shareholder) **Kimberly Workowski, MD**, Nothing to disclose

